# Short-Term Rapamycin Preconditioning Diminishes Therapeutic Efficacy of Human Adipose-Derived Stem Cells in a Murine Model of Multiple Sclerosis

**DOI:** 10.3390/cells9102218

**Published:** 2020-09-30

**Authors:** Rachel M. Wise, Mark A. A. Harrison, Brianne N. Sullivan, Sara Al-Ghadban, Sarah J. Aleman, Amber T. Vinluan, Emily R. Monaco, Umberto M. Donato, India A. Pursell, Bruce A. Bunnell

**Affiliations:** 1 Neuroscience Program, Tulane Brain Institute, Tulane University School of Science & Engineering, New Orleans, LA 70118, USA; rwise@tulane.edu (R.M.W.); mharri26@tulane.edu (M.A.A.H.); bsulliv7@tulane.edu (B.N.S.); saleman@tulane.edu (S.J.A.); avinluan@tulane.edu (A.T.V.); emonaco@tulane.edu (E.R.M.); udonato1@tulane.edu (U.M.D.); 2 Center for Stem Cell Research & Regenerative Medicine, Tulane University School of Medicine, New Orleans, LA 70112, USA; sara.al-ghadban@unthsc.edu (S.A.-G.); ipursell@tulane.edu (I.A.P.); 3 Department of Microbiology, Immunology and Genetics, University of North Texas Health Science Center, Fort Worth, TX 76107, USA; 4 Department of Pharmacology, Tulane University School of Medicine, New Orleans, LA 70112, USA

**Keywords:** adipose tissue-derived stem cells (ASCs), multiple sclerosis (MS), experimental autoimmune encephalomyelitis (EAE), Rapamycin, immunomodulation, inflammation, demyelination

## Abstract

Human adipose-derived stem cells (ASCs) show immense promise for treating inflammatory diseases, attributed primarily to their potent paracrine signaling. Previous investigations demonstrated that short-term Rapamycin preconditioning of bone marrow-derived stem cells (BMSCs) elevated secretion of prostaglandin E2, a pleiotropic molecule with therapeutic effects in the experimental autoimmune encephalomyelitis (EAE) model of multiple sclerosis (MS), and enhanced immunosuppressive capacity in vitro. However, this has yet to be examined in ASCs. The present study examined the therapeutic potential of short-term Rapamycin-preconditioned ASCs in the EAE model. Animals were treated at peak disease with control ASCs (EAE-ASCs), Rapa-preconditioned ASCs (EAE-Rapa-ASCs), or vehicle control (EAE). Results show that EAE-ASCs improved clinical disease scores and elevated intact myelin compared to both EAE and EAE-Rapa-ASC animals. These results correlated with augmented CD4^+^ T helper (T_h_) and T regulatory (T_reg_) cell populations in the spinal cord, and increased gene expression of interleukin-10 (IL-10), an anti-inflammatory cytokine. Conversely, EAE-Rapa-ASC mice showed no improvement in clinical disease scores, reduced myelin levels, and significantly less T_h_ and T_reg_ cells in the spinal cord. These findings suggest that short-term Rapamycin preconditioning reduces the therapeutic efficacy of ASCs when applied to late-stage EAE.

## 1. Introduction

Multiple sclerosis (MS) is an inflammation-driven autoimmune disease caused by aberrant activation and infiltration of peripheral immune cells into the central nervous system (CNS), and subsequent destruction of myelin-producing oligodendrocytes [1]. MS affects an estimated 2.3 million people worldwide, with over 85% of patients presenting with the relapsing-remitting form of the disease [2,3]. Many of these patients develop secondary progressive MS—at which point, disease progressively worsens without periods of remission, and most available therapies lose their efficacy [4]. The current treatments for MS are primarily disease-modifying and immunosuppressive drugs that target the inflammation, but not the demyelination and neurodegenerative damage that cause the primary symptoms of MS [5]. It is, therefore, imperative to explore safe and effective regenerative therapies to address these significant limitations. The experimental autoimmune encephalomyelitis (EAE) animal model is the best characterized for the study of pathogenic mechanisms in MS. In the EAE mouse, the induction of myelin-specific autoreactive T cells results in a cascade of CNS inflammation, demyelinating lesion formation, and ascending motor function deterioration that peaks 2 to 3 weeks post-immunization [6].

Adipose tissue-derived stem cells (ASCs) are characterized by their multipotency and self-renewal capacity. However, it is their potent immunoregulatory properties that make them a promising therapeutic intervention for autoimmune, inflammatory, and neurodegenerative diseases [7]. ASCs have significant benefits over other tissue sources of mesenchymal stem cells (MSCs), such as bone marrow, due to their ease of harvest, higher stem cell yield, enhanced secretion of immune-modifying factors, and reduced immunogenicity post-transplant [8,9]. Our lab has repeatedly demonstrated the beneficial effects of ASC treatment in the EAE model of MS. We have shown that ASCs suppress proliferation of type 1 T helper (Th_1_) cells, promote T regulatory (T_reg_) cells and alternatively-activated macrophages (M2), dampen pro-inflammatory cytokine production, reduce CNS infiltration and demyelinating lesions, and improve overall disease outcomes [10,11,12,13]. The mechanisms driving the ability of ASCs to slow or reverse disease progression in EAE are not fully defined, but there is substantial evidence that the primary benefit of ASC therapy is their paracrine modulation of immune cell populations rather than their engraftment into CNS tissues and direct replacement of oligodendrocytes. Melief et al. demonstrated the superior secretory activity of ASCs compared to BMSCs, which is associated with better suppression of PBMC proliferation and differentiation into mature dendritic cells [14]. This secretory activity is also correlated with the therapeutic activity of ASCs in the EAE model, as inhibition of secreted immunomodulatory factors including PGE2 mitigates immunoregulatory capacity [15].

One study by Payne and collaborators demonstrated the superior homing ability of human ASCs to the inflamed CNS in the EAE mouse model, which was correlated with improved disease scores, reduced CNS infiltration and demyelination in ASC-treated animals [16]. However, an investigation by Constantin et al. showed that while a small subset of IV-injected GFP-labeled ASCs were observed in spinal cord tissues up to 6 weeks post-treatment, less than 2% co-expressed mature glial markers, making it unlikely that engraftment and transdifferentiation of ASCs contributes significantly to their therapeutic effects [17]. Another report showed persistence of injected cells in the CNS of EAE mice up to 25 days post-injection but did not assess engraftment or differentiation [18]. Additionally, this study based these assumptions on human transcript levels in the EAE rat brain, not on the visualization of cells. Thus, most evidence of ASC therapy in EAE suggests that their primary benefit derives from their homing to sites of inflammation, modifying innate and adaptive immune cells through paracrine activity, and changing the populations that then infiltrate the CNS and determine the course of disease.

Despite substantial preclinical success, translation to human trials faces numerous obstacles due to limited and variable ASC immunosuppression in the post-transplant pathological environment. Furthermore, donor characteristics such as advanced age or obesity status negate the therapeutic effect of ASCs, and strategies to restore their efficacy would reduce donor-to-donor variability and drastically widen the potential donor pool [13,19,20].

To address these challenges, in vitro preconditioning strategies have been developed to improve both the survival and immune response of MSCs to stressful stimuli [21]. One of the most successful strategies is the immunosuppressive drug Rapamycin (Rapa), which is FDA approved for treatment of several cancers, both alone and in combination with chemotherapeutic agents [22]. The preconditioning of MSCs with Rapa results in substantial improvement of their immunoregulatory function in animal models of graft vs. host disease and cutaneous wound healing [23,24]. Moreover, Rapa exposure has demonstrated an effective reversal of MSC senescence [25,26,27], resiliency to apoptosis-inducing stimuli [28], and robust enrichment of immunomodulatory function [29,30,31]. Furthermore, short-term Rapa exposure caused an increase in both cyclooxygenase-2 (COX2) and prostaglandin-E2 (PGE2) in bone marrow-derived mesenchymal stem cells (BMSCs) in vitro [30]. PGE2, the synthetic byproduct of COX2 enzymatic activity on arachidonic acid, is typically upregulated in response to inflammatory conditions and modulates innate and adaptive immune cells [32,33]. This upregulation correlated with enhanced suppression of proliferation of peripheral blood mononuclear cells (PBMCs) and splenocytes [30]. However, it has yet to be determined whether the beneficial effects of Rapa seen in vitro can be extrapolated to ASC therapy for EAE.

Recent in vivo evidence from a study of BMSC administration in EAE mice suggests that one BMSC-derived signaling molecule, PGE2, may constitute a substantial part of the therapeutic efficacy of MSC therapy in EAE [34]. Matysiak et al. found that PGE2 inhibition following BMSC treatment significantly reduced the therapeutic effect [34]. Intriguingly, PGE2 has exhibited both beneficial and harmful roles in EAE progression. In early preclinical phases, PGE2 receptor expression and secretion are enhanced in monocytes and macrophages. This is associated with elevated inflammatory T cell activation and pro-inflammatory cytokine production [35]. In the later prodromal phase of EAE, expression of PGE2 receptors is increased on T cells, which demonstrate enhanced migration, expression of matrix metallopeptidase 9 (MMP9), and invasion of the lumbar spinal cord [35]. Additionally, PGE2 and its receptors are elevated in active lesions in EAE and MS [36], and knockout studies show that PGE2 activity is crucial for the development of EAE [35,37]. However, it has been demonstrated that PGE2 can also suppress disease progression by protecting or restoring the integrity of the blood–brain barrier (BBB) if administered during a critical window [37]. Importantly, the immunological consequences of PGE2 overexpression during active EAE remain unexplored.

Faced with conflicting evidence on the contribution of PGE2 to EAE pathophysiology, and the reported elevation of PGE2 following short-term Rapa in ASCs, the present study investigates the consequences of this preconditioning strategy in the EAE model of CNS inflammation. Following disease induction, treatments with either ASCs (EAE-ASC), Rapa-preconditioned ASCs (EAE-Rapa-ASC), or vehicle control (EAE) were compared for their ability to improve symptoms and performance on the rotarod task, mitigate CNS cellular infiltration and damaged myelin, and modify T cell populations in the lymphoid organs and the spinal cord. The data demonstrate that while ASCs can improve most of the examined disease outcomes when applied at days post-induction (DPI) 20, Rapa-ASCs proved to be no more effective than vehicle treatment. These findings, though unexpected, highlight the necessity of in vivo assessment of novel treatment strategies. These findings suggest that further investigation of Rapa-ASCs earlier in EAE may yield greater insight into the dynamic role of PGE2 in disease pathogenesis.

## 2. Materials and Methods

### 2.1. Induction of EAE with Myelin Oligodendrocyte Glycoprotein (MOG)_35–55_ Peptide

Reagents for EAE induction were prepared by diluting MOG_35–55_ peptide (2 mg/mL; Cat #: AS-60130-5; AnaSpec, Fremont, CA, USA) in UltraPure™ DNase/RNase-Free distilled water (ThermoFisher, Waltham, MA, USA) and emulsifying with equal parts of Complete Freund’s Adjuvant (BD Biosciences, Franklin Lakes, NJ, USA) containing 8 mg/mL *Mycobacterium tuberculosis* H37RA (Cat #: 231131; BD Biosciences, San Jose, CA, USA) by passage through glass Luer-Lok syringes and a micro-emulsifying needle for 45 min. The emulsion was then transferred to 1 mL Luer-Lok syringes with 27G ½” needles. Pertussis toxin was diluted in UltraPure™ water (2 ng/μL; Cat #: 181; List Biologicals Laboratories, Campbell, CA, USA) and transferred to syringes as described above. Female 6–8-week-old C57Bl/6 mice (Charles River Laboratories, Wilmington, MA, USA) were anesthetized using 5% isoflurane gas then given bilateral subcutaneous flank injections of 100 μL MOG emulsion near the base of the tail (200 μL total per mouse). Concurrently, mice were given a single intraperitoneal (IP) injection of 100 μL pertussis toxin. Mice received a second IP injection of 100 μL pertussis toxin 48 h later to complete the EAE induction process. Sham-induced control mice received equivalent injections of Hank’s balanced salt solution (HBSS; ThermoFisher, Waltham, MA, USA). All animal procedures were authorized by the Institutional Animal Care and Use Committee at Tulane University and followed state and federal National Institute of Health’s animal welfare guidelines. Mice were given food pellets and water ad libitum. Using a standard clinical rating scale, mice were scored daily for disease progression by blinded researchers starting at 1 day post-induction (DPI) and going through DPI 30. Briefly, mice were given a score from 0 to 5: 0 no detectable signs of disease; 1, tail atony with abnormal gait; 2, hind limb weakness; 3, partial hind limb paralysis; 4, complete hind limb paralysis; 5, moribund or dead.

### 2.2. Rotarod Analysis

To assess balance and coordination in vehicle-treated (EAE, *n* = 5), ASC-treated (EAE-ASC, *n* = 5) and Rapa-preconditioned ASC-treated (EAE- Rapa-ASC, *n* = 6) mice, the Roatmex-5 rotarod system (Columbus Instruments, Columbus, OH, USA) for small rodents was used as previously described by others [38,39]. Each experimental mouse was subjected to three training sessions from DPI 3 to 5. Following that, the mice were tested weekly at a fixed rotational speed of 4 rpm for a maximum time of 2 min. The latency to fall across three consecutive trials was recorded and group mean ± SEM was reported.

### 2.3. Cells and Cell Culture

Primary human ASCs were purchased from LaCell LLC (New Orleans, LA, USA). Individual ASC cell lines were fully characterized individually prior to being pooled [19,20,40,41,42]. ASCs from 5 healthy donors were pooled and expanded in complete culture medium (CCM) consisting of Minimum Essential Medium alpha (Cat #: 12561; Gibco, Grand Island, NY, USA) supplemented with 10% heat-inactivated Hyclone characterized fetal bovine serum (FBS, Cat #: SH30396.03; ThermoFisher, Waltham, MA, USA), and 1% Penicillin-Streptomycin (Cat #: 15140122; 10,000 U/mL, ThermoFisher, Waltham, MA, USA) in a humidified, 5% CO2 incubator. Media was changed every 2–3 days until cells achieved 70–80% confluence. ASCs were used at passage 5 for the experiments.

### 2.4. Preparation and Injection of Cells

Based on our previous EAE studies, DPI 20 was chosen for late-stage treatment [11]. On DPI 20, cultured ASCs were washed with 1XPBS (ThermoFisher, Waltham, MA, USA) then treated for 4 h with either control CCM (ASCs) or Rapamycin-supplemented CCM (Rapa-ASCs; 500 nM; Cat #: 553211; Millipore Sigma, Burlington, MA, USA). Cells were then washed with 1XPBS, harvested with 0.25% trypsin/1 mM EDTA (Cat #: 25200056; ThermoFisher, Waltham, MA, USA), and live cells were counted using a trypan blue exclusion assay. Finally, 1 × 10^6^ ASCs or Rapa-ASCs were resuspended in 100 μL HBSS and transferred to 1 mL Luer-Lok syringes with 27G, ½” needles for IP injections as previously described [11,19,20]. Mice with a clinical score of 2 or greater on DPI 20 were randomly assigned to treatment groups and received 100 μL IP injections of 1 × 10^6^: ASCs (EAE-ASC, *n* = 5), Rapa-ASCs (EAE-Rapa-ASC, *n* = 6), or HBSS (EAE, *n* = 5) for vehicle control.

### 2.5. Tissue Harvest and Processing

EAE mice were euthanized by CO_2_ asphyxiation and the spleens and spinal cords of each mouse were harvested. Lumbar sections of spinal cords (L3–L6) were removed and stored at room temperature (RT) in neutral buffered formalin for subsequent paraffin embedding. Remaining spinal cord tissue was homogenized in Qiazol lysis reagent (Cat #: 79306; Qiagen, Germantown, MD, USA) and immediately stored at −80 °C for future experiments. EAE spleens were mechanically dissociated by passing through a 100 µm cell strainer using the blunt ends of syringes into a 50 mL conical tube and centrifuged at 2000 rpm for 5 min to pellet. The cells were then incubated with red blood cell lysis (company info) for 5 min at RT. Splenocytes were then washed with PBS, counted using trypan blue exclusion assay, and processed for flow cytometric analysis or stored in Qiazol lysis reagent at −80 °C for subsequent RNA isolation.

### 2.6. Flow Cytometric Staining and Analysis

Splenocytes were counted and resuspended in 1XPBS containing the cell viability indicator GhostDye 780 (Cat #: 13-0865; Tonbo Biosciences, San Diego, CA, USA) and incubated for 30 min at 4 °C. Cells were then washed twice with flow staining buffer containing 1% bovine serum albumin (BSA; Sigma-Aldrich, St. Louis, MO, USA), spun down and resuspended in flow staining buffer to a final concentration of 1 × 10^6^ cells/mL. Next, cells were stained with fluorescently conjugated anti-mouse antibodies against CD3 (Cat #: 11-0032-82, ThermoFisher, Waltham, MA, USA), CD4 (Cat #: 56004282, Fisher Scientific, Lenexa, KS, USA), and CD8 (Cat #: 12008183, eBioscience, San Diego, CA, USA). Samples requiring intracellular staining were incubated in a fixation/permeabilization solution (Cat #: 88-8824-00; ThermoFisher, Waltham, MA, USA) and stained with a fluorescently conjugated anti-mouse antibody against intracellular FOXP3 (Cat #: 12400-31; SouthernBiotech, Birmingham, AL, USA). All other samples were washed and fixed using 1% paraformaldehyde. Samples were stored at 4 °C until flow cytometric analysis could be performed using a Gallios Flow Cytometer (Beckman Coulter). A minimum of 1 × 10^4^ events per sample were captured and analyzed with Kaluza Analysis 2.1 software (Beckman Coulter).

### 2.7. RNA Isolation and Quantitative Reverse-Transcription PCR (qRT-PCR)

Homogenized EAE spleens and spinal cords stored in Qiazol at −80 °C were thawed and RNA was extracted from each sample using the Qiagen RNeasy Plus mini kit (Cat #: 74136, Qiagen, Germantown, MD, USA). A total of 1 µg of mRNA per sample was synthesized into cDNA using the Applied Bioscience High-Capacity cDNA Reverse Transcription kit (Cat #: 4368814, ThermoFisher, Waltham, MA, USA). qRT-PCR was performed with SsoAdvanced Universal SYBR Green Supermix (Cat #: 1725271, Bio-Rad, Hercules, CA, USA). Mouse-specific, exon-spanning primers were designed using the Primer-BLAST online tool4 and synthesized by Integrated DNA Technologies (Coralville, IA, USA). Forward and reverse primer sequences used for qRT-PCR are listed in Table 1. All reactions were performed in duplicate. Analysis was completed using the 2^−ΔΔCt^ method to calculate the relative fold-change in transcript expression after normalization to the reference gene, β-actin. Data for all groups were normalized to the vehicle control group (EAE) for relative quantification of mRNA expression levels.

### 2.8. Histological Analysis of Spinal Cords

Formalin-fixed lumbar spinal cords were paraffin embedded, cut into 5 μm thick sections, and mounted on microscope slides. Sections were stained with hematoxylin (Cat #: 7231; ThermoFisher, Waltham, MA, USA) and eosin (Cat #: 7111; ThermoFisher, Waltham, MA, USA) to assess cellular infiltration, or Luxol fast blue (LFB; cat# IW-3005, IHC World, Ellicott City, MD, USA) to determine myelin content. Slides were imaged using a Zeiss Axio Scan.Z1 slide scanner (Carl Zeiss Microscopy; Gottingen, Germany), and brightfield images were analyzed using the ZEN3.1 image analysis software (Carl Zeiss Microscopy). Four animals were randomly selected from each group, and at least 6 spinal cord sections across a 400 μm region were analyzed by a blinded researcher. Myelin content and cellular infiltration were calculated as the number of positive pixels/total number of pixels and reported as % total area.

### 2.9. Statistical Analysis

All data are presented as the mean ± SEM. GraphPad PRISM 8 (GraphPad; San Diego, CA, USA) was used to perform all statistical analyses. Results of single time points were compared using one-way analysis of variance (ANOVA), and results across several time points were compared using a mixed effects model of repeated measures ANOVA followed by a Tukey’s post-hoc test. Asterisks (*) denote statistical significance between the HBSS vehicle-treated control group and the ASC-treated or Rapa-ASC-treated groups: * *p* < 0.05; ** *p* < 0.01; and *** *p* < 0.001. Pound signs (#) denote statistical significance between the ASC-treated and Rapa-ASC-treated groups: # *p* < 0.05; ## *p* < 0.01; and ### *p* < 0.001.

## 3. Results

### 3.1. ASCs, but Not Rapa-ASCs, Modestly Improved Rotarod Performance in EAE Mice

**Figure 1:**
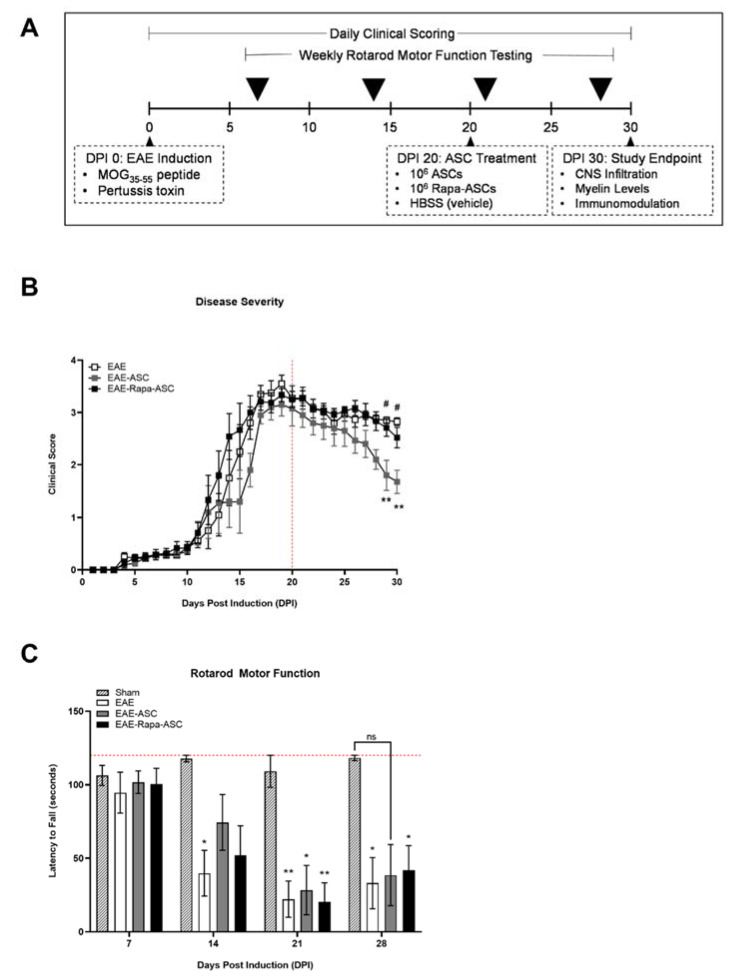
Rapa-ASCs do not reduce disease severity when administered at late-stage EAE. (**A**) Experimental design illustrating the timeline of EAE induction and schedule of clinical and behavioral assessments. (**B**) Severity of disease progression for each group over the course of 30 days, as determined by traditional clinical scoring system. (**C**) Balance and motor coordination evaluation with a fixed speed rotarod performance test. Reported as latency to fall. All data are presented as the mean ± SEM. Statistical analysis was performed using a mixed effects model of repeated measures analysis of variance (ANOVA) and Tukey’s post-hoc multiple comparisons test. Statistical differences between EAE and EAE-ASC are marked with * *p* < 0.05; ** *p* < 0.01. Statistical differences between EAE-ASC and EAE-Rapa-ASC are marked with # *p* < 0.05. Abbreviations: EAE, experimental autoimmune encephalomyelitis; Rapa, Rapamycin; MOG, myelin oligodendrocyte glycoprotein.

Human ASCs have shown considerable therapeutic ability in the EAE mouse model of MS [10,15,18,19,20,43,44]. Thus, we investigated whether a novel Rapa preconditioning approach enhanced or inhibited their immunomodulatory capacity. After the induction of EAE, mice were scored daily for disease progression using a common clinical rating scale. At DPI 20, a time point representing the height of disease severity, animals were randomly assigned to receive either control ASCs (EAE-ASC, *n* = 5), 4-h Rapa-preconditioned ASCs (EAE-Rapa-ASC, *n* = 6) or vehicle control treatment (EAE, *n* = 5) (Figure 1A). We demonstrate that the ASC-treated group showed a marked improvement in disease scores compared to both vehicle control and Rapa-ASC groups by DPI 30, indicating the successful reduction in disease severity (Figure 1B).

Motor coordination and balance were analyzed weekly by fixed speed rotarod testing, to assess functional recovery. One week before treatment (DPI 14) and before assignment to groups randomly, all EAE animals showed deficits in rotarod performance compared to sham-induced animals (Figure 1C). Twenty-four hours after ASC treatment, on DPI 21, the EAE-ASC group resulted in the most significant improvement on the rotarod task demonstrated by the increased latency to fall. All animals began to show signs of improvement by DPI 28.

### 3.2. Reduced Myelin Content of the CNS in ASC-, but Not Rapa-ASC-Treated EAE Mice

**Figure 2:**
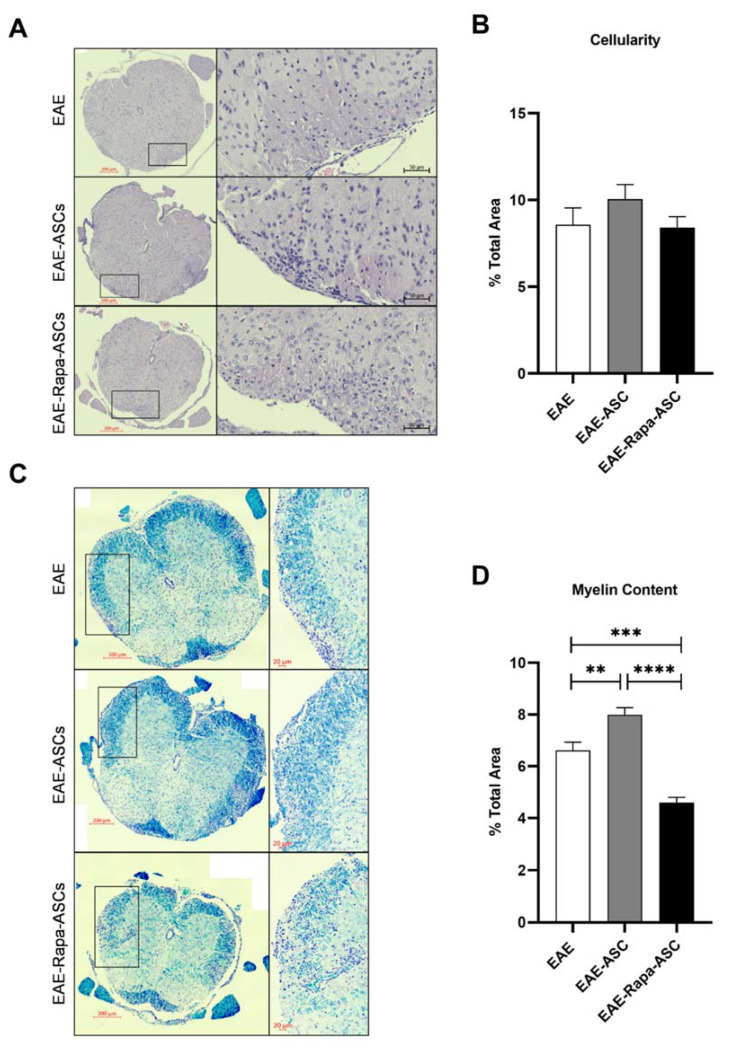
Rapa-ASCs result in reduced intact myelin when administered during late-stage EAE. Representative images of (**A**) hematoxylin and eosin-stained and (**C**) Luxol fast blue-stained lumbar spinal cord sections from vehicle-treated EAE (EAE), ASC-treated EAE (EAE-ASC) and Rapa-ASC-treated EAE (EAE-Rapa-ASC) mice. Quantitative comparison of (**B**) cellular infiltration and (**D**) myelin content in spinal cord sections between each group (*n* = 4). Quantitative data are represented as the number of positive pixels divided by the total pixels of the section. Statistical analysis was performed using a mixed effects model of repeated measures analysis of variance (ANOVA) and Tukey’s post-hoc multiple comparisons test. Statistical differences between the mean ± SEM of EAE and EAE-ASC are marked with ** *p* < 0.01; *** *p* < 0.001; and **** *p*<0.0001.

One of the most critical pathological hallmarks of MS and EAE is the extravasation of peripheral immune cells into the CNS and subsequent destruction of myelin in the spinal cord. In this study, histological analysis of EAE lumbar spinal cord sections was used to assess cellular infiltration and demyelination. As demonstrated by hematoxylin staining of cell nuclei, there was no quantifiable difference in spinal cord cellularity in EAE mice receiving ASC treatments compared with vehicle-treated EAE mice (Figure 2A,B). Conversely, intact myelin levels, measured by dark blue LFB staining, were significantly enhanced in EAE-ASC mice compared to EAE controls (Figure 2C,D). Thus, ASC treatment at DPI 20 is capable of either preventing further myelin damage or repairing the extant damage. Furthermore, Rapa-ASC treatment resulted in substantially reduced myelin levels compared to both vehicle and EAE-ASC mice, suggesting that Rapa-preconditioned ASCs may exacerbate rather than mitigate the demyelination seen in the EAE spinal cord.

### 3.3. ASC, but Not Rapa-ASC, Treatment Resulted in Elevated T Cell Marker in the CNS of EAE Mice

**Figure 3:**
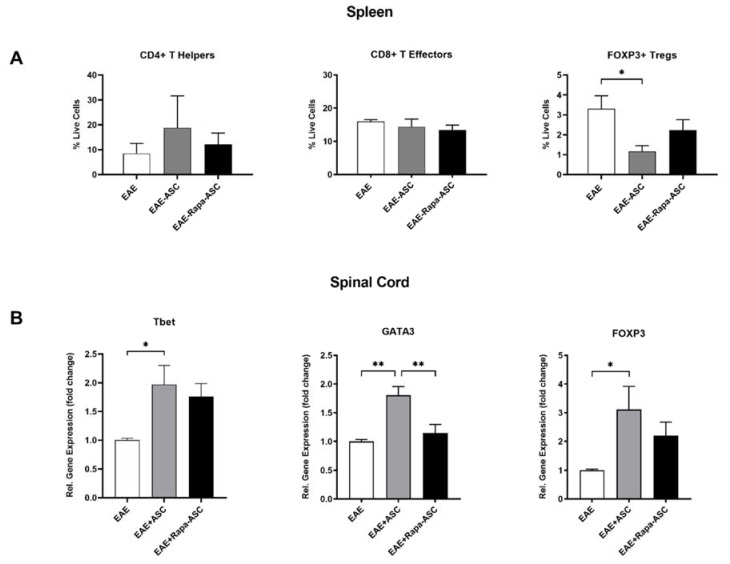
ASCs and Rapa-ASCs differentially regulate immune cell populations in the spleen and spinal cord 10 days post-treatment. (**A**) Cells isolated from spleens of vehicle-treated EAE (*n* = 5), ASC-treated EAE (*n* = 5), and Rapa-ASC-treated EAE (*n* = 6) mice were analyzed for canonical T cell markers with flow cytometry and represented as percentage of live cells. (**B**) Gene expression analysis of key T cell transcription factors in whole EAE spinal cords was performed using RT-qPCR. Data are presented as the mean ± SEM. Data are normalized to the housekeeping gene β-actin and represented as the relative fold change over vehicle-treated EAE controls. Statistical analysis was performed using a one-way analysis of variance (ANOVA) and Tukey’s post-hoc multiple comparisons test. Statistical differences between groups are marked with * *p* < 0.05; ** *p* < 0.01. FOXP3, Forkhead Box P3; Tbet, T-box expressed in T cells; GATA3, GATA Binding Protein 3.

The ability of ASC and Rapa-ASC therapy to modify both peripheral and CNS-permeated T cell populations was examined in EAE mice at DPI 30. As demonstrated by flow cytometric analysis of splenocytes, EAE-ASC mice exhibited a trend of increased CD4^+^ Th cells and a decrease in CD4^+^/FOXP3^+^ T_reg_ cells (Figure 3A). Neither ASCs nor Rapa-ASCs significantly altered CD4^+^/CD8^+^ T effector (T_eff_) populations in the spleen (Figure 3A). Gene expression levels of T cell transcription factors (TFs) were robustly increased in the spinal cord after ASC, but not Rapa-ASC, treatment (Figure 3B). T-box expressed in T cells (Tbet) and GATA Binding Protein 3 (GATA3) are essential transcription factors for the differentiation of the pro-inflammatory Th_1_ and anti-inflammatory Th_2_ T helper cells, respectively [45]. Tbet and GATA3 gene expression were upregulated in spinal cord tissue from EAE-ASC mice compared to EAE vehicle-treated controls. Forkhead Box P3 (FOXP3) is a key TF that drives the differentiation of T_regs_, which play crucial roles in quieting inflammation and promoting tissue regeneration [46]. Following ASC treatment, FOXP3 was increased more than 3-fold in the spinal cord compared to vehicle-treated EAE mice. While Rapa-preconditioned ASCs show trends toward enhanced Tbet and FOXP3 expression, this fails to reach statistical significance.

### 3.4. IL-10 Gene Expression is Significantly Increased with ASC- and Rapa-ASC-Treated EAE Mice

**Figure 4:**
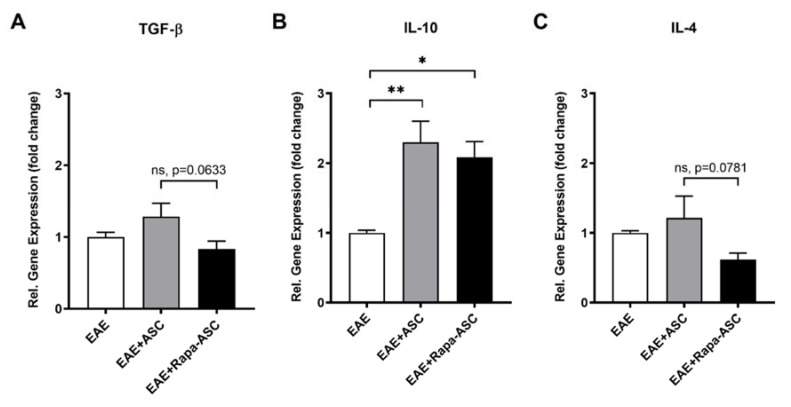
Gene expression analysis of T helper cell cytokines in the EAE spinal cord is altered by ASC treatments. Whole EAE spinal cords were analyzed using RT-qPCR for the T helper cell-derived cytokines TGF-β (**A**), IL-10 (**B**), and IL-4 (**C**). Data are presented as the mean ± SEM. Data are normalized to the housekeeping gene β-actin and represented as the relative fold change ± SEM over vehicle-treated EAE controls. Statistical analysis was performed using a one-way analysis of variance (ANOVA) and Tukey’s post-hoc multiple comparisons test. Statistical differences between groups are marked with * *p* < 0.05; ** *p* < 0.01. TGF-β, transforming growth factor-beta; IL-10, interleukin-10; IL-4, interleukin-4.

We next examined gene expression of common T cell-derived cytokines that contribute to the anti-inflammatory and pro-regenerative activity of both T_regs_ and Th_2_ cells (Figure 4). T_regs_ produce high levels of transforming growth factor-beta (TGF-β) and interleukin-10 (IL-10), which both suppress differentiation of Th_1_ cells from naïve CD4^+^ T cells and suppress antigen presenting cell function [47]. At the transcriptional level, IL-10 showed a significant increase in EAE-ASC and to a lesser extent in EAE-Rapa-ASC mice as compared to EAE controls. No change, however, has been detected in TGF-β or IL-4 gene expression. Activated Th_2_ cells produce high levels of IL-4, which can polarize macrophages and microglia towards the anti-inflammatory, M2 phenotype. After the administration of Rapa-ASCs to established EAE, a trend towards reduced IL-4 gene expression was observed in the spinal cord when compared with the control ASC treatment. Altogether, these findings indicate that ASC treatment is more effective than Rapa-ASCs at upregulating immune mediators in EAE.

## 4. Discussion

Human ASCs have demonstrated robust therapeutic efficacy in preclinical models of MS [10,15,18,19,20,43,44], primarily through their immunoregulation of innate and adaptive immune cells [48,49]. However, clinical success with ASCs faces many challenges, including post-transplant apoptosis and weakened immunomodulatory potency [50]. Previous work suggests that short-term, but not long-term, inhibition of the mammalian target of rapamycin (mTOR) activity intensifies mesenchymal stem cell immunomodulation. In BMSCs, short-term Rapa results in elevated production of immune-modifying factors increased immunosuppression of PBMC or splenocyte proliferation, and protection against apoptosis following exposure to ischemia or oxygen-glucose deprivation [28,51]. In the present study, we examined the effect of short-term mTOR inhibition with Rapamycin on the immunomodulatory capacity of ASCs in the EAE mouse model of MS. Our results demonstrate that control ASCs exhibited significant therapeutic benefits when administered at DPI 20, as indicated by improved clinical scores, modestly better rotarod performance, and increased myelin levels in the lumbar spinal cord compared to vehicle-treated EAE mice. Surprisingly, EAE-Rapa-ASC mice failed to show measurable improvements in clinical scores, rotarod ability, or myelin levels, suggesting that Rapa preconditioning diminished the ASC immunoregulatory ability in vivo.

Prostaglandin E2, a pleiotropic molecule synthesized from arachidonic acid by COX2, has been implicated in the immunoregulatory actions of mesenchymal stem cell therapies in the EAE mouse. Anderson and colleagues have shown that mouse ASCs were able to suppress the pathophysiology and progression of EAE through robust inhibition of dendritic cell maturation and subsequent T cell proliferation [15]. Inhibition of COX2 abolished this effect on dendritic cells in vitro, indicating that PGE2 may be primarily responsible for the in vivo results [15]. Another study by Matysiak and collaborators demonstrated a therapeutic role for PGE2, but not IL-10 or TGF-β, in the EAE model. They administered a COX2 inhibitor in parallel with BMSC treatment, which resulted in reduced therapeutic efficacy [34]. While both groups have investigated PGE2 deprivation on stem cell therapies in the EAE model, none have examined whether elevated PGE2 enhances therapeutic benefits.

In vitro evidence from BMSCs showed short-term Rapa-mediated elevation of PGE2 signaling resulted in enhanced suppression of PBMC and splenocyte proliferation, suggesting that overexpressed PGE2 may amplify the benefit seen in EAE mice [30]. Therefore, the present study applied short-term Rapa preconditioning to ASCs and examined changes to therapeutic efficacy in EAE. The results indicate that control ASCs robustly reduced symptom severity and modestly improved performance on a fixed speed rotarod test. However, Rapa-ASCs demonstrated no similar improvements as compared to vehicle-treated EAE mice, indicating a loss of therapeutic potency. Timing of ASC administration may explain these findings, as others have shown that PGE2 plays temporally distinct roles in EAE pathogenesis and progression. A report by Esaki et al. illustrated a critical window of therapeutic benefit for PGE2 actions which may be mediated by its ability to prevent BBB permeabilization [37]. Their findings may highlight an unexpected benefit to the current investigation. Our laboratory previously demonstrated that human ASC therapy is highly effective prophylactically and therapeutically when administered at peak disease severity, but is less efficacious at suppressing disease progression if applied at DPI 8, a time point correlative to T cell extravasation into CNS and emergence of initial motor symptoms [52,53,54,55]. Thus, if short-term Rapa elevate ASC-derived PGE2 and PGE2 prevents BBB breakdown, future investigations may determine whether Rapa-ASC treatment at this early pathogenic stage may inhibit extravasation of T cells.

T_regs_ play a crucial role in suppressing demyelination in EAE and are a critical factor of any therapeutic strategy. Selective depletion of T_regs_ from the CNS results in rampant T effector proliferation, pro-inflammatory cytokine production, and increased disease severity. T_regs_ have also exhibited robust suppression of antigen presenting cells, limiting their ability to activate T effector cells [56]. These indicate a significant role for T_regs_ in modulation of T effector functions [57]. Furthermore, ASC treatment of EAE mice at DPI 15 has demonstrated that enhancement of T_reg_ markers accompanies improved disease scores and reduced demyelination in the spinal cord [18,44]. The current study revealed changes to T_reg_ populations both in the periphery and in the spinal cord of EAE mice that received ASC treatment, but not those that received Rapa-ASC treatment, at DPI 20. As revealed by flow cytometric analysis of splenocytes, CD4^+^/FOXP3^+^ T_regs_ are considerably reduced after ASC treatment, but not after Rapa-ASC or vehicle treatment. This correlates with a large transcriptional upregulation of the essential T_reg_ transcription factor, FOXP3, in the EAE spinal cord suggesting that peripheral T_regs_ may be mobilized by ASC but not Rapa-ASC or vehicle treatment. T_reg_ production of the cytokines TGF-β and IL-10 are essential for their suppression of pathogenic autoreactive T cells [58]. In the spinal cord, both ASC and Rapa-ASC treatment resulted in elevated gene expression levels of IL-10, with ASCs resulting in the greatest elevation compared to vehicle-treated controls. However, neither treatment significantly altered TGF-β transcripts in the spinal cord compared to vehicle controls. The data indicate that while ASC treatment at DPI 20 may enhance spinal cord markers of T_regs_, Rapa-ASCs exhibit a reduced capacity to promote T_reg_ functions in the CNS which correlates with a loss of symptomatic and rotarod improvement.

Following activation by antigen-presenting cells, CD4^+^ T cells also differentiate into a variety of T helper phenotypes depending upon the cytokine milieu [59]. Th_1_ cells are classically pro-inflammatory and require the transcription factor Tbet for differentiation. In MS, Th_1_ cells are highly enriched in blood and CSF and secrete encephalitogenic cytokines that drive disease progression [60]. Th_2_ cells are considered anti-inflammatory and differentiate due to the activity of the transcription factor GATA3. In EAE, Th_2_ cells have displayed both pathogenic and immunoregulatory activities. Th_2_ cytokines can redirect autoreactive Th_17_ cell trafficking away from the CNS [61] and indirectly suppress Th_1_ cell function by regulating antigen-presenting cell activity [62]. However, they can also induce or exacerbate EAE in adoptive transfer models [63]. In EAE, both early and late therapeutic application of ASCs demonstrated immunomodulation of T cell cytokines, showing preferential promotion of IL-4 and IL-10 with concomitant suppression of IFN-γ [18]. The current study interrogated Th_1_ and Th_2_ cell subsets to determine the immunomodulatory action of ASCs and Rapa-ASCs in the spleen and spinal cord. Flow cytometric analysis of splenocytes revealed a trend towards increased CD4^+^ Th cells in the ASCs, but not the Rapa-ASCs, treated spleen, suggesting that control ASCs have stronger immunoregulatory potential than Rapa-ASCs. A parallel effect was observed in the spinal cord, with both Tbet and GATA3 gene expression showing more significant upregulation in EAE-ASC than EAE-Rapa-ASC mice. However, the levels of IL-4 mRNA, an anti-inflammatory cytokine that both induces and is produced by Th_2_ cells, were not significantly different between groups.

Our data show that Rapa-ASCs resulted in significantly lower levels of Th_2_ cell differentiation markers, and a trend in lower Th_1_ and T_reg_ markers compared to control ASCs. Furthermore, examination of the T_reg_ and Th_2_ cell-associated cytokines TGF-β1, IL-10, and IL-4 revealed a trend toward lowered cytokine expression in the spinal cord of EAE-Rapa-ASC mice compared to control ASCs. Taken together, these findings suggest that the ability of Rapa-ASCs to modulate T cell differentiation and cytokine production after transplant into the EAE pathological environment is dampened compared to control ASCs. This may at least partially explain why intact myelin is lower in this group than both ASC and vehicle-treated controls. The balance of T cells is critically important for MS and EAE intervention, as was revealed by an investigation of global CD4^+^ T cell depletion in MS patients which resulted in no clinical benefit [64]. Based on our transcriptional data, we propose that control ASCs, but not Rapa-ASCs, may shift the balance in favor of T_reg_ and Th_2_ cells over Th_1_ cells and promote a CNS environment more favorable to oligodendrocyte protection, repair, or regeneration.

Th_1_-derived cytokines including IL-2, IL-12, and IFNγ promote further proliferation of Th_1_ cells and inhibit proliferation of Th_2_ cells [65,66]. Th_2_-derived cytokines including IL-4 and IL-10, promote expansion of Th_2_ cells and inhibit the proliferation and function of Th_1_ cells [65,66]. T_regs_ suppress T effector and M1 macrophage activity, and their depletion in EAE mice resulted in elevated T_eff_ and macrophage proliferation and exacerbated disease severity [57].

While the focus of the current work was on T cell populations and associated cytokines, these play a crucial regulatory role in the activation of antigen-presenting cells such as infiltrating macrophages and resident microglia. Th_1_-derived cytokines activate M1-type microglia [67], while Th_2_-derived cytokines activate M2-type macrophages and microglia [68,69]. M1 activated macrophages and microglia have been found in early and active demyelinating lesions, suggesting their direct role in tissue destruction [70,71,72]. Furthermore, these cells produce pro-inflammatory cytokines and chemokines that activate astrocytes, impair the BBB, and recruit further encephalitogenic immune cells from the periphery [73]. Conversely, M2 macrophages and microglia have been implicated in remyelination in EAE [74]. Therefore, future investigations into macrophage and microglial phenotypes and associated neuroinflammatory cytokines may shed light on the worsened myelin loss in the EAE-Rapa-ASC spinal cord compared to both EAE and EAE-ASC animals.

Short-term Rapa exposure was hypothesized to enhance the therapeutic efficacy of ASCs. However, results from this study indicate that this preconditioning strategy eliminated their robust immunomodulatory effect. Rapa-ASCs were unable to preferentially promote T_reg_ and Th cell subsets, transcription of the anti-inflammatory cytokine IL-10, the increase in myelin levels, and improvement of rotarod performance when compared to ASC treatment. These findings emphasize that while novel preconditioning approaches may show promise in vitro or in direct co-culture studies, it is imperative to examine them in the context of physiologically relevant disease models. The disease course of MS and EAE is dynamic, with constantly shifting immune populations. Future studies should be conducted to determine whether this loss of therapeutic potential in Rapa-ASCs is limited to their application to advanced-stage disease, or whether there may be a critical window for which elevated PGE2 secretion is beneficial. The augmentation of ASC immunoregulatory capacity remains a significant obstacle for translational success. This study reveals that short-term Rapa may not be a viable approach for established EAE and should be interrogated for earlier disease applications. This work highlights the importance of investigating novel treatment strategies in animal models of disease, as in vitro work cannot effectively recapitulate the complexities of the in vivo pathological microenvironment. 

## Figures and Tables

**Table 1:** Primer Sequences.

Gene	Forward (5′–3′)	Reverse (5′–3′)
*Beta-actin*	GTGGGCCGCCCTAGGCACCA	TTAGCACGCACTGTAGTTTCTC
*TGF-β*	CGTCAGACATTCGGGAAGCA	TGCCGTACAACTCCAGTGAC
*IL-10*	GCTCTTGCACTACCAAAGCC	CTGCTGATCCTCATGCCAGT
*IL-4*	GGTCTCAACCCCCAGCTAGT	GCCGATGATCTCTCTCAAGTGAT
*Tbet*	CACTAAGCAAGGACGGCGAA	TAATGGCTTGTGGGCTCCAG
*GATA3*	TGTCTGCGAACACTGAGCTG	CGATCACCTGAGTAGCAAGGA
*FOXP3*	CCCATCCCCAGGAGTCTTG	ACCATGACTAGGGGCACTGTA
